# Association Between the *Tips From Former Smokers* Campaign and Smoking Cessation Among Adults, United States, 2012–2018

**DOI:** 10.5888/pcd17.200052

**Published:** 2020-08-27

**Authors:** Rebecca Murphy-Hoefer, Kevin C. Davis, Brian A. King, Diane Beistle, Robert Rodes, Corinne Graffunder

**Affiliations:** 1 Office on Smoking and Health, National Center for Chronic Disease Prevention and Health Promotion, Centers for Disease Control and Prevention, Atlanta, Georgia; 2 Center for Health Policy Science and Tobacco Research, RTI International, Research Triangle Park, North Carolina

## Abstract

In 2012, the Centers for Disease Control and Prevention (CDC) launched the national *Tips From Former Smokers* (*Tips*) campaign to encourage people who smoke to quit by showing real-life heath consequences of tobacco use and promoting evidence-based resources for quitting. To assess the campaign’s impact on quit attempts and sustained-quit estimates (ie, quits lasting ≥6 mos), CDC analyzed data from a nationally representative longitudinal survey of US adults who smoke cigarettes, aged 18 years or older in 2012–2018. The *Tips* campaign was associated with an estimated 16.4 million quit attempts and 1,005,419 sustained quits. Continued implementation of cessation campaigns, including the *Tips* campaign, could accelerate progress toward reducing rates of smoking-related diseases and death.

**Summary**

**What is already known on this topic?**

The Centers for Disease Control and Prevention’s* Tips From Former Smokers *(*Tips*) campaign is associated with increased quit attempts among specific populations of people who smoke, including African Americans, pregnant women, people with mental health conditions, and those with lower educational attainment. The campaign increases calls to smoker quitlines and visits to the *Tips* website and other cessation resources.

**What is added by this report?**

During 2012–2018, the *Tips* campaign was associated with an estimated 16.4 million quit attempts and more than 1 million sustained quits among US adults.

**What are the implications for public health practice?**

Mass media campaigns, such as the *Tips *campaign, can increase smoking quit attempts and sustained quits as part of a comprehensive approach to reducing smoking-related disease and premature death in the United States.

**MEDSCAPE CME**

In support of improving patient care, this activity has been planned and implemented by Medscape, LLC and *Preventing Chronic Disease*. Medscape, LLC is jointly accredited by the Accreditation Council for Continuing Medical Education (ACCME), the Accreditation Council for Pharmacy Education (ACPE), and the American Nurses Credentialing Center (ANCC), to provide continuing education for the healthcare team.

Medscape, LLC designates this Journal-based CME activity for a maximum of 1.00 AMA PRA Category 1 Credit(s)™. Physicians should claim only the credit commensurate with the extent of their participation in the activity.

Successful completion of this CME activity, which includes participation in the evaluation component, enables the participant to earn up to 1.0 MOC points in the American Board of Internal Medicine’s (ABIM) Maintenance of Certification (MOC) program. Participants will earn MOC points equivalent to the amount of CME credits claimed for the activity. It is the CME activity provider’s responsibility to submit participant completion information to ACCME for the purpose of granting ABIM MOC credit.

Release date: August 27, 2020; Expiration date: August 27, 2021

**Learning Objectives**

Upon completion of this activity, participants will be able to:

1. Distinguish the ratio of persons who die from smoking-related illness to persons living with smoking-related illness
2. Assess the efficacy of the *Tips From Former Smokers* **^®^** program
3. Analyze characteristics associated with higher rates of attempts to quit smoking

**EDITOR**

Camille Martin, RD

Editor

Preventing Chronic Disease

Disclosure: Camille Martin, RD, has disclosed no relevant financial relationships.

**CME AUTHOR**

Charles P. Vega, MD

Health Sciences Clinical Professor of Family Medicine

University of California, Irvine, School of Medicine

Irvine, California 

Disclosure: Charles P. Vega, MD, has disclosed the following relevant financial relationships:

Served as an advisor or consultant for: Johnson & Johnson Pharmaceutical Research & Development, LLC; GlaxoSmithKline

Served as a speaker or a member of a speakers bureau for: Genentech, GlaxoSmithKline

**AUTHORS**

Rebecca Murphy-Hoefer, PhD, MPH

Office on Smoking and Health

National Center for Chronic Disease Prevention and Health Promotion

Centers for Disease Control and Prevention (CDC)

Atlanta, Georgia

Disclosure: Rebecca Murphy-Hoefer, PhD, MPH, has disclosed no relevant financial relationships.

Kevin C. Davis, MA

Center for Health Policy Science and Tobacco Research

RTI International

Research Triangle Park, North Carolina

Disclosure: Kevin C. Davis, MA, has disclosed no relevant financial relationships.

Brian A. King, PhD, MPH

Office on Smoking and Health

National Center for Chronic Disease Prevention and Health Promotion

Centers for Disease Control and Prevention (CDC)

Atlanta, Georgia

Disclosure: Brian A. King, PhD, MPH, has disclosed no relevant financial relationships.

Diane Beistle, BA

Office on Smoking and Health

National Center for Chronic Disease Prevention and Health Promotion

Centers for Disease Control and Prevention (CDC)

Atlanta, Georgia

Disclosure: Diane Beistle, BA, has disclosed no relevant financial relationships.

Robert Rodes, MS, MBA

Office on Smoking and Health

National Center for Chronic Disease Prevention and Health Promotion

Centers for Disease Control and Prevention (CDC)

Atlanta, Georgia

Disclosure: Robert Rodes, MS, MBA, has disclosed the following relevant financial relationships:

Other: Spouse is employed by Elekta, Inc.

Corinne Graffunder, DrPH, MPH

Office on Smoking and Health

National Center for Chronic Disease Prevention and Health Promotion

Centers for Disease Control and Prevention (CDC)

Atlanta, Georgia

Disclosure: Corinne Graffunder, DrPH, MPH, has disclosed no relevant financial relationships.

## Objective

Cigarette smoking remains the leading cause of preventable death in the United States (1). For every person who dies because of cigarette smoking, at least 30 people live with a serious smoking-related illness (1). Evidence-based media campaigns can increase tobacco cessation, increase use of cessation resources such as quitlines, and change tobacco-related social norms (2,3). This study aimed to determine the 7-year impact of the *Tips From Former Smokers* (*Tips*) campaign on population-level smoking cessation by measuring cumulative campaign-associated quit attempts and sustained quit estimates, accounting for smoking relapse.

## Methods

The Centers for Disease Control and Prevention (CDC) collected data from the KnowledgePanel (www.knpanel.com) (KP), an ongoing national online survey of adults in the United States. KP recruitment is conducted through random sampling of US household mailing addresses, and respondents are followed over time, allowing for participation in multiple survey waves. Current cigarette smokers are defined as people who smoked at least 100 cigarettes in their lifetime and who smoked every day or some days at the time of survey. We included data from the 2012–2018 waves of this survey (N = 35,275 observations on 9,653 unique current smokers) to assess the impact of *Tips* campaign exposure on quit attempts and sustained quit estimates.

We used a geography-based quasi-experimental design that relates variation in *Tips* campaign exposure across media markets and time to individual quit attempt behaviors across time. Quit attempts in the past 3 months among current cigarette smokers were assessed by asking, “During the past 3 months, how many times have you stopped smoking for 1 day or longer because you were trying to quit smoking cigarettes for good?” We created an indicator variable for having made at least 1 quit attempt in the past 3 months. *Tips* campaign exposure was determined by calculating past 3-month cumulative campaign television gross ratings points (GRPs, a measure of market-level campaign dose) and merging them with individual survey responses based on respondents’ media market of residence and survey date. We used logistic regression to relate self-reported quit attempts in the past 3 months to GRPs (3). The model controlled for age, sex, race/ethnicity, education level, annual household income, presence of chronic physical or mental health conditions, tobacco surveys taken in the past year, presence of children in the household, presence of others who smoke cigarettes in the household, cigarette smoking prevalence in the respondent’s television market, state fixed effects, and a linear time trend to control for secular trends over time. Model results were used to estimate the predicted quit attempt rate differential between observed doses of zero GRPs (ie, no campaign) and the average quarterly *Tips* campaign dose of 1,200 GRPs from 2012 to 2018 (ie, matching CDC recommendations on GRP dose) (2). The quit attempt rate differential was then multiplied by the yearly adult smoker population to create an initial estimate of total campaign-attributable quit attempts for each year during 2012–2018. Finally, the year-specific projections of campaign-attributable quit attempts were adjusted to account for the number of quarters the campaign was on the air in each year.

Sustained quit estimates were calculated using the estimated proportion of campaign-attributable quit attempters who remained abstinent from smoking at 6-month follow-up. On the basis of survey timing and resources, we were able to estimate sustained quitting during 4 of the 7 years of data in the analysis. Sustained quit rates averaged 7.2% in the available data. Because our sample was not designed to measure longer-term relapse, we used literature-based estimates (4,5) to calculate approximate relapse (15.3%) for 1 year after the initial 6 months of cigarette abstinence.

## Results

The *Tips* campaign was correlated with increased odds of a quit attempt in the past 3 months (odds ratio = 1.19; 95% confidence interval [CI], 1.11–1.27) (Table 1). An average of 1,200 GRPs per quarter translated into a 3.9 (95% CI, 3.4–4.3) percentage point increase in quit attempts per quarter during 2012–2018. Past 3-month quit attempt rates ranged from 32.5% in the absence of the campaign (0 GRPs) to 39.7% (4,000 GRPs) during the 2012–2018 campaigns (4,000 GRPs). Approximately 16.4 million quit attempts and an estimated 1,005,419 sustained quits lasting at least 1 year (95% CI, 876,519–1,108,539) were associated with *Tips* during 2012–2018 (Table 2). Sustained quit estimates ranged from 103,729 in 2012 to 188,577 in 2017.

**Table 1:** Association Between Making a Quit Attempt in the Past 3 Months and Select Characteristics, *Tips From Former Smokers* Campaign, United States, 2012–2018

Model Covariate^a^	OR (95% CI)
**Total campaign mass media market GRPs, past 3 months (square root functional form)**	1.19 (1.11–1.27)
**Age**	0.98 (0.98–0.99)
**Sex**	
Female	1 [Reference]
Male	0.87 (0.78–0.97)
**Race/ethnicity**	
White	1 [Reference]
Black	1.55 (1.29–1.86)
Hispanic	1.90 (1.59–2.26)
Other	1.37 (1.08–1.75)
**Education**	
Less than high school	1 [Reference]
High school diploma	1.08 (0.91–1.29)
Some college	1.28 (1.08–1.52)
Bachelor's degree or higher	1.69 (1.38–2.06)
**Annual household income, $**	
<20,000	1 [Reference]
20,000–49,999	0.88 (0.78–1.00)
50,000–99,999	0.97 (0.84–1.12)
≥100,000	0.80 (0.67–0.97)
**Chronic condition**	1 [Reference]
Physical	1.27 (1.14–1.42)
Mental	1.08 (0.97–1.20)
**Tobacco surveys past year**	0.93 (0.90–0.96)
**Child in household**	1.25 (1.12–1.40)
**Smoker in household**	0.56 (0.51–0.62)
**Smoking prevalence (mass media market level)**	0.99 (0.96–1.02)
**Linear time**	0.997 (0.99–1.00)
**No. of model observations**	35,275

Abbreviations: CI, confidence interval; GRPs, gross rating points; OR, odds ratio.

a Model includes covariates for state fixed effects (not shown).

**Table 2:** Estimated Campaign Cumulative Impact on Sustained Quits, *Tips From Former Smokers* Campaign, United States, 2012–2018

Campaign Year	Dates On Air	Number of Quarters Campaign on Air	Estimated Campaign-Associated Quit Attempts (n = 16,440,928)	Estimated Campaign-Associated Sustained Quits^a^ (n = 1,005,419)
2012	March 19–June 19	1.00	1,696,214	103,729
2013	March 4–June 17	1.16	1,964,772	120,152
2014	February 3–April 6; July 7–September 7	1.50	2,436,389	148,994
2015	March 30–August 16	1.49	2,198,523	134,447
2016	January 25–June 12	1.53	2,385,108	145,858
2017	January 9–July 30	2.22	3,083,677	188,577
2018	April 23–October 14	1.92	2,676,245	163,662

a Assuming a 15.3% relapse rate.

## Discussion

During 2012–2018, the *Tips* campaign contributed to 16.4 million quit attempts and more than 1 million estimated sustained quits. These results are consistent with previous evaluations of the *Tips* campaign that have shown significant campaign effects on quit attempts and sustained quit estimates (eg, 1.6 million and 100,000 in 2012 (6); 1.83 million and 104,000 in 2014 (7); and 9 million and 522,000 in 2012–2015 (8), respectively). Additionally, the impact of *Tips *on quit attempts was recently supported with a study using data from the Behavioral Risk Factor Surveillance System (9).

Prior studies have also reported the impact of the *Tips* campaign on quit attempts among specific populations, including African Americans, pregnant women, people with mental health conditions, and those with less educational attainment (10). The campaign has also been associated with cessation-related outcomes, such as increased calls to 1–800-QUIT-NOW (6) and 1–855-DEJELO-YA (a national portal that routes Spanish-speaking callers to Spanish-language services from callers’ state quitlines) (11), and visits to the *Tips* campaign website and other cessation resources (10).

These findings are subject to at least 2 limitations. The analysis used an average campaign effect estimated from 2012 to 2018. Although this effect may vary across years, research does not indicate significant variation in campaign effects over time (3). Another limitation is that we measured only television exposure and not other campaign channels such as radio, digital media, or billboards. Therefore, the estimated campaign effects may be conservative if total campaign exposure was underestimated.

In summary, the *Tips* campaign led to an estimated 16.4 million quit attempts and more than 1 million estimated sustained quits during 2012–2018, demonstrating that public health campaigns can be effective when they are based on scientific evidence and are of sufficient intensity and duration (2).

## Post-Test Information

To obtain credit, you should first read the journal article. After reading the article, you should be able to answer the following, related, multiple-choice questions. To complete the questions (with a minimum 75% passing score) and earn continuing medical education (CME) credit, please go to http://www.medscape.org/journal/pcd. Credit cannot be obtained for tests completed on paper, although you may use the worksheet below to keep a record of your answers.

You must be a registered user on http://www.medscape.org. If you are not registered on http://www.medscape.org, please click on the “Register” link on the right hand side of the website.

Only one answer is correct for each question. Once you successfully answer all post-test questions, you will be able to view and/or print your certificate. For questions regarding this activity, contact the accredited provider, CME@medscape.net. For technical assistance, contact CME@medscape.net. American Medical Association’s Physician’s Recognition Award (AMA PRA) credits are accepted in the US as evidence of participation in CME activities. For further information on this award, please go to https://www.ama-assn.org. The AMA has determined that physicians not licensed in the US who participate in this CME activity are eligible for AMA PRA Category 1 Credits™. Through agreements that the AMA has made with agencies in some countries, AMA PRA credit may be acceptable as evidence of participation in CME activities. If you are not licensed in the US, please complete the questions online, print the AMA PRA CME credit certificate, and present it to your national medical association for review.

## Post-Test Questions

### Study Title: Association Between the *Tips From Former Smokers* Campaign and Smoking Cessation Among Adults, United States, 2012–2018

#### CME Questions

1. You are seeing a 35-year-old man who has smoked approximately one-half of a pack per day of cigarettes since he was 20 years old. He has no complaints or chronic illnesses and needs a physical examination to start work as a commercial truck driver. He says he is not interested in smoking cessation now because "I'm not dying anytime soon." What can you quote him regarding the ratio of number of deaths related to cigarette smoking to number of individuals living with smoking-related illness?
  - 1:4
  - 1:12
  - 1:30
  - 1:120
2. You tell this patient about the *Tips From Former Smokers*® campaign to provide more information and motivation to quit smoking. In the current study, which of the following outcomes was associated with the *Tips From Former Smokers* campaign?
  - Neither quit attempts nor sustained smoking cessation
  - Quit attempts but not sustained smoking cessation
  - Sustained smoking cessation but not quit attempts
  - Both quit attempts and sustained smoking cessation
3. Which of the following other variables was associated with a higher rate of attempts to quit smoking in the current study?
  - Black and Hispanic race/ethnicity
  - Male sex
  - Higher income
  - Having another smoker in the household

## References

[1] US Department of Health and Human Services. The health consequences of smoking — 50 years of progress: a report of the Surgeon General. Atlanta (GA): US Department of Health and Human Services, Centers for Disease Control and Prevention, National Center for Chronic Disease Prevention and Health Promotion, Office on Smoking and Health; 2014.

[2] Centers for Disease Control and Prevention. Best practices for comprehensive tobacco control programs — 2014. Atlanta (GA): US Department of Health and Human Services, Centers for Disease Control and Prevention, National Center for Chronic Disease Prevention and Health Promotion, Office on Smoking and Health; 2014.

[3] Davis KC , Patel D , Shafer P , Duke J , Glover-Kudon R , Ridgeway W , Association between media doses of the Tips From Former Smokers campaign and cessation behaviors and intentions to quit among cigarette smokers, 2012–2015. Health Educ Behav 2018;45(1):52–60. 10.1177/1090198117709316 28497703 PMC6193261

[4] Dai H , Leventhal AM . Association of electronic cigarette vaping and subsequent smoking relapse among former smokers. Drug Alcohol Depend 2019;199:10–7. 10.1016/j.drugalcdep.2019.01.043 30978519 PMC6743978

[5] Herd N , Borland R , Hyland A . Predictors of smoking relapse by duration of abstinence: findings from the International Tobacco Control (ITC) Four Country Survey. Addiction 2009;104(12):2088–99. 10.1111/j.1360-0443.2009.02732.x 19922574 PMC4517970

[6] McAfee T , Davis KC , Alexander RL Jr , Pechacek TF , Bunnell R . Effect of the first federally funded US antismoking national media campaign. Lancet 2013;382(9909):2003–11. 10.1016/S0140-6736(13)61686-4 24029166

[7] Neff LJ , Patel D , Davis K , Ridgeway W , Shafer P , Cox S . Evaluation of the national Tips From Former Smokers campaign: the 2014 longitudinal cohort. Prev Chronic Dis 2016;13:150056. 10.5888/pcd13.150556 27010845 PMC4807436

[8] Murphy-Hoefer R , Davis KC , Beistle D , King BA , Duke J , Rodes R , Impact of the Tips From Former Smokers campaign on population-level smoking cessation, 2012–2015. Prev Chronic Dis 2018;15:180051. 10.5888/pcd15.180051 29862960 PMC5985905

[9] Davis KC , Murphy-Hoefer R , Levine B , King BA , Hu S , Rodes R . Evidence of the impact of the Tips From Former Smokers Campaign: results From the Behavioral Risk Factor Surveillance System. Prev Chronic Dis 2019;16:190110. 10.5888/pcd16.190110 31603406 PMC6795073

[10] Centers for Disease Control and Prevention. Tips impact and results. https://www.cdc.gov/tobacco/campaign/tips/about/impact/campaign-impact-results.html. Accessed June 10, 2020.

[11] Zhang L , Babb S , Johns M , Mann N , Thompson J , Shaikh A , Impact of US antismoking TV ads on Spanish-language quitline calls. Am J Prev Med 2018;55(4):480–7. 10.1016/j.amepre.2018.05.025 30241616

